# Begelomab for severe refractory dermatomyositis: A case report

**DOI:** 10.1097/MD.0000000000024372

**Published:** 2021-03-05

**Authors:** Rebecca De Lorenzo, Clara Sciorati, Antonella Monno, Silvia Cavalli, Francesco Bonomi, Stefano Tronci, Stefano Previtali, Patrizia Rovere-Querini

**Affiliations:** a Division of Immunology, Transplantation and Infectious diseases, IRCCS Ospedale San Raffaele; b Università Vita-Salute San Raffaele; c Institute of Experimental Neurology (InSpe), Division of Neuroscience, IRCCS Ospedale San Raffaele, Milan, Italy.

**Keywords:** begelomab, case report, dermatomyositis, dipeptidyl peptidase-4/cluster of differentiation 26, idiopathic inflammatory myopathy

## Abstract

**Rationale:**

Severe refractory idiopathic inflammatory myopathy (IIM) represents a challenge for the clinician. The lack of efficacy of available tools reflects our incomplete insight into the molecular events sustaining the inflammatory tissue damage in these patients. We present the first case of refractory IIM treated with anti-dipeptidyl peptidase-4 (DPP-4)/cluster of differentiation 26 (CD26) monoclonal antibody.

**Patient concerns:**

A 55-year old man presented with proximal muscle weakness, diffuse erythematous skin lesions which rapidly evolved into ulcerations, dysphagia and dysphonia.

**Diagnosis:**

Increased serum creatine kinase levels and histological findings at muscle and skin biopsies were compatible with the diagnosis of dermatomyositis (DM). Several lines of treatment failed to control the disease including steroids, mycophenolate mofetil, tacrolimus, intravenous immunoglobulins and rituximab. Despite therapy, the patient also had recurrent intestinal vasculitis causing bowel perforation. Concurrently, DPP-4/CD26 expression in the patient's skin and skeletal muscle was observed.

**Interventions:**

The patient was treated with begelomab, a murine immunoglobulin G2b monoclonal antibody against DPP-4/CD26.

**Outcomes:**

Dysphagia, skin lesions and intestinal vasculitis resolved and the patient experienced a significant improvement of his quality of life.

**Conclusion:**

Blockade of DPP-4/CD26, which is expressed on T cells and mediates T cell activation and function, is safe and might be effective in patients with refractory DM.

## 1 Introduction

Dermatomyositis (DM) is an idiopathic inflammatory myopathy (IIM) characterized by skin lesions and skeletal muscle weakness.^[1]^ A breakdown of immune-tolerance towards specific antigens, driven by a combination of environmental factors and genetic predisposition, leads to the perpetuation of tissue inflammation and damage. This may translate into clinically evident organ dysfunction. Depending on the tissue tropism of the inflammatory response, organs other than skin and muscles are affected. Vasculitis of the gastrointestinal tract has been described in juvenile DM patients,^[2–4]^ while in adults is anecdotal.^[5,6]^ Management of severe multi-organ forms of disease is arduous, and complete disease remission with currently available treatments is often unattainable.

T lymphocytes are key players in DM pathogenesis,^[7,8]^ and vascular damage and insufficiency downstream their activation cause architectural and functional alterations in DM muscle tissue.^[9]^ The signals involved in disrupting hometostasis of T cells in tissues of patients with DM are poorly characterized.^[8,10–12]^ Dipeptidyl peptidase-4 (DPP-4), also known as cluster of differentiation 26 (CD26), is a candidate for such a role. It is a membrane glycoprotein endowed with enzymatic activity,^[13,14]^ expressed by cells that play a direct or indirect role in the pathogenesis of DM including hematopoietc cells, endothelial cells and activated fibroblasts involved in wound healing.^[15–20]^ It promotes effector T cell activation by sustaining selected signal transduction pathways^[21]^ specifically promoting the clonal expansion of Th17 and Th1 cells,^[22]^ known to have a role in IIM pathogenesis.^[23–26]^ DPP-4/CD26^high^ CD4^+^ T cells respond maximally to recall antigens, migrate to inflammatory tissues and efficiently activate B cells for antibody production.^[16,27]^ In contrast, it is not or very poorly expressed by T regulatory cells.^[28]^ Moreover, DPP-4/CD26 expression in different tissues influences glucose control and systemic inflammation.^[20]^ Since DPP-4/CD26 is highly expressed on T cells that infiltrate the gut and skin, and its integrity is required for migration through the endothelial barrier, CD26 has represented a target for the treatment of graft versus host disease (GvHD) with monoclonal antibodies.^[29]^ Here we verified that DPP-4/CD26 was indeed expressed in the skin and skeletal muscle of a patient with a severe life-threating DM characterized by multiple organ involvement and refractory to multiple conventional immunosuppressive therapies. The patient has benefitted from treatment with Begelomab, a murine immunoglobulin G (IgG) 2b monoclonal antibody against DPP-4/CD26.

## 2 Case presentation

**Figure 1:**
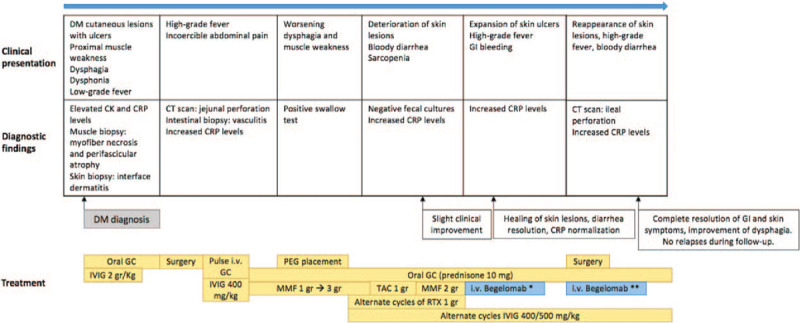
Disease and treatments over time. Infectious events and temporary treatment suspensions due to surgery or infections are not depicted. ^∗^ At the dosage of 4 mg/m^2^ daily for 5 consecutive days. ^∗∗^ Two cycles were performed at a distance of 3 weeks one from the other: the first one between the onset of skin symptoms and ileal perforation, and the other soon after ileal resection. The second one included a maintenance regimen of 11 infusions (one infusion of 4 mg/m^2^ daily) every other day. DM = dermatomyositis, CRP = C reactive protein, GC = glucocorticoids, IVIG = intravenous immunoglobulins, MMF = mycophenolate mofetil, GI = gastrointestinal tract, TAC = tacrolimus.

**Figure 2:**
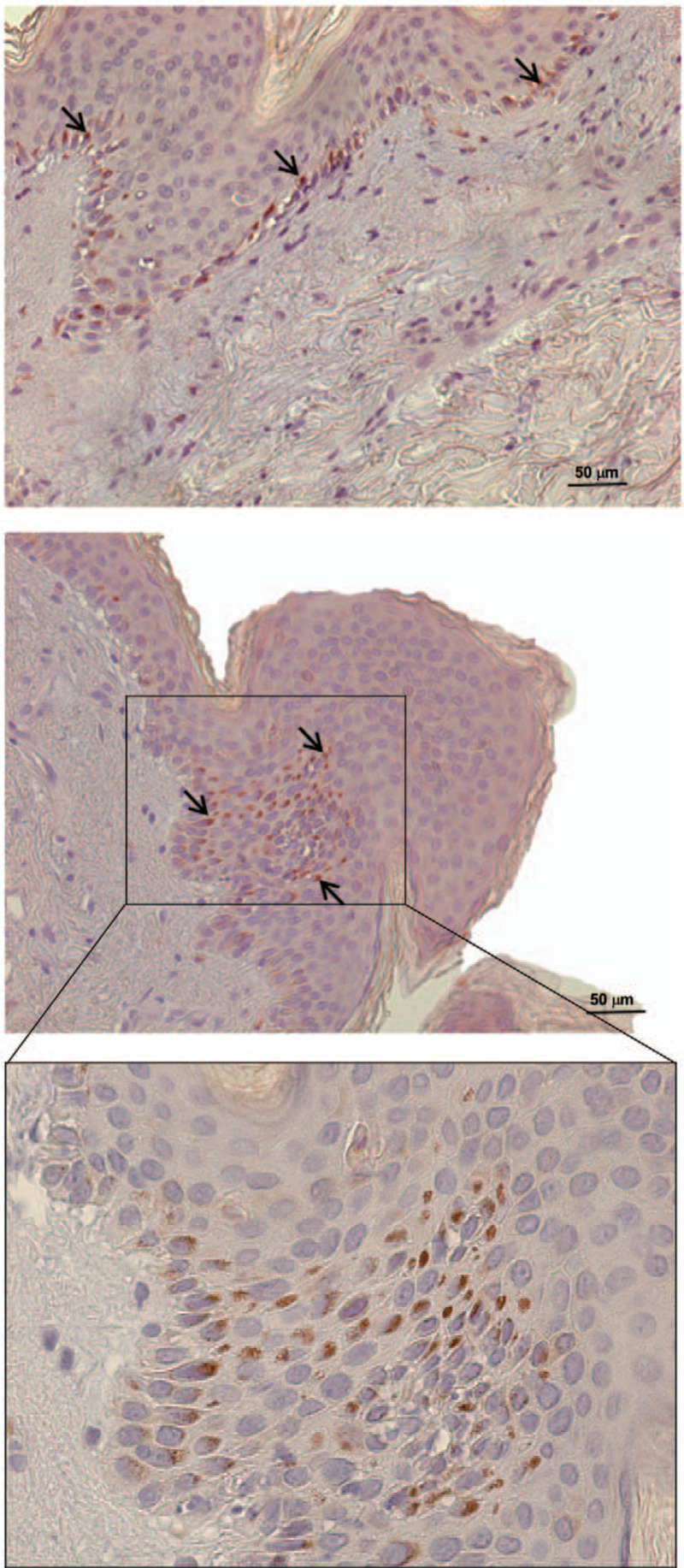
CD26 expression in the skin. Skin was analyzed through immunohistochemistry for DPP-4/CD26 expression (brown). Infiltrating leucocytes and cells of the basal epidermal layer express the antigen (arrows).

**Figure 3:**
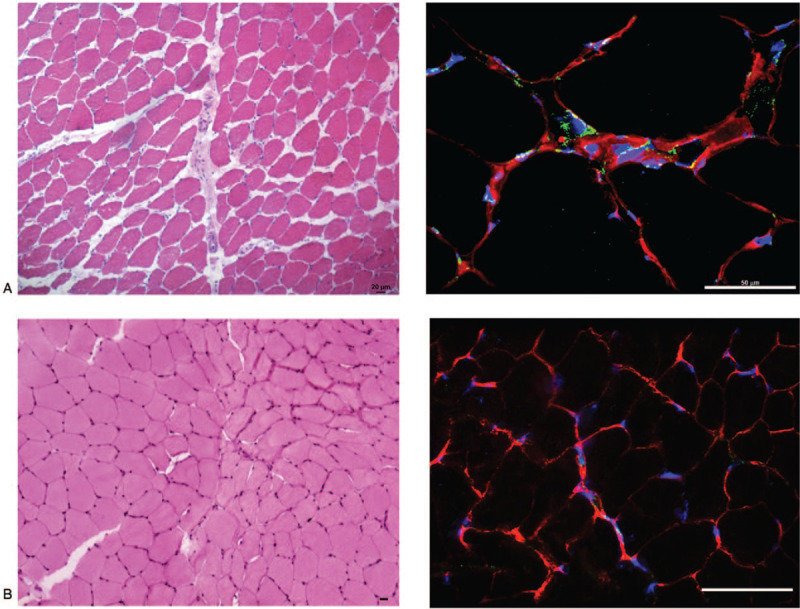
CD26 expression in the skeletal muscle. Muscles from the patient (A) and a healthy control (B) were analyzed through Hematoxylin and eosin (H&E, left) and immunofluorescence (green color, right) for DPP-4/CD26 expression. DAPI (blue) was used to counterstain nuclei and laminin expression (red) to identify fiber boundaries. DPP-4/CD26 is preferentially expressed in the interstitial and perivascular spaces.

We describe the case of a 55-year old man with a history of acute myocardial infarction causing secondary dilative cardiomyopathy, under statin treatment since the age of 41, with no family history for autoimmune diseases. He developed persisting low-grade fever, proximal symmetrical muscle weakness and a diffuse erythematous rash of chest, neck and upper back, as wells as violaceous papules at metacarpophalangeal and interphalangeal joints and other bony prominences. Few weeks later, swallowing difficulty, dysphonia and dyspnea due to oral cavity edema appeared, cutaneous lesions developed into ulcers, while muscle weakness became severe and disabling. Blood tests revealed increased creatine kinase levels (1085 U/L) and slightly elevated C-reactive protein concentration (7.7 mg/dl). Myositis-specific and -associated autoantibodies were negative. Muscle biopsy revealed myofiber necrosis, degeneration and regeneration of myofibers with variation in myofiber size, and perifascicular atrophy. Histopathological findings of skin biopsy comprised interface dermatitis with focal, granular deposits of IgG and complement component 3 at the dermo-epidermal junction. In light of the clinical presentation and the above mentioned diagnostic outcomes, the patient was diagnosed with DM. An oncological screening excluded the presence of malignancy. Statins were immediately discontinued. The patient started methylprednisolone 0.7 mg/Kg daily to be slowly tapered, and received intravenous immunoglobulins (IVIG, 2 g/Kg over 5 consecutive days) with clinical benefit. One month later, however, he experienced the abrupt onset of high-grade fever and incoercible abdominal pain. A computed tomography scan with oral contrast revealed jejunal perforation and an emergency laparotomy followed by segmental jejunal resection with latero-lateral anastomosis was performed. Multiple macroscopic ischemic lesions and ulcers were found intraoperatively within the intestinal mucosa, and the histopathological analysis indicated intestinal vasculitis. One week later the patient underwent a revision surgery because of anastomosis dehiscence and subsequent enteric fistula. After clinical stabilization, the patient was treated with pulses of glucocorticoids (over 3 consecutive days) and a cycle of IVIG 400 mg/kg. The therapy was continued with prednisone 10 mg and mycophenolate mofetil (MMF) 1 gr daily, which was increased progressively up to 3 gr daily over 3 weeks. Three months after MMF introduction, the clinical response was not satisfactory. Due to significant dysphagia, a percutaneous endoscopic gastrostomy had to be placed and muscle weakness was so debilitating that the patient needed a wheelchair. After 2 more months, while under MMF therapy, the patient experienced a further worsening of cutaneous lesions and the sudden onset of bloody diarrhea, requiring repeated blood transfusions. In-hospital diagnostics excluded an infectious etiology of intestinal symptoms and Rituximab (RTX) was administered. Unfortunately, enterococcal sepsis developed. Immunosuppressive treatment was thus interrupted and antibiotics initiated. At the end of antimicrobial therapy, due to the poor clinical response to RTX, MMF was reintroduced together with low-dose prednisone. Despite some improvement in intestinal symptoms, control of skin manifestations was not achieved and MMF was substituted with Tacrolimus (Fig. 1). In the following 2 months, the patient also received IVIG and RTX while continuing glucocorticoids, with no clinical benefit. Ulcerated skin lesions expanded in size and number, especially on the face, genitals and arms, and significant sarcopenia became evident. Tacrolimus was suspended and MMF reintroduced again. Two other cycles of IVIG were administered in the following 2 months. One month after the last IVIG administration, high-grade fever, extensive gastrointestinal bleeding and skin and mucosal ulcerations reappeared. In consideration of the inadequate response to multiple immunosuppressive regimens, we reasoned that an alternative strategy should be found. Given the clinical features of the patient, resembling cutaneous and intestinal involvement of GvHD, the skin and muscle biopsies were analyzed by immunocytochemistry and immunofluorescence for DPP-4/CD26 expression. The antigen was expressed in both tissues (Figs. 2 and 3).

**Figure 4:**
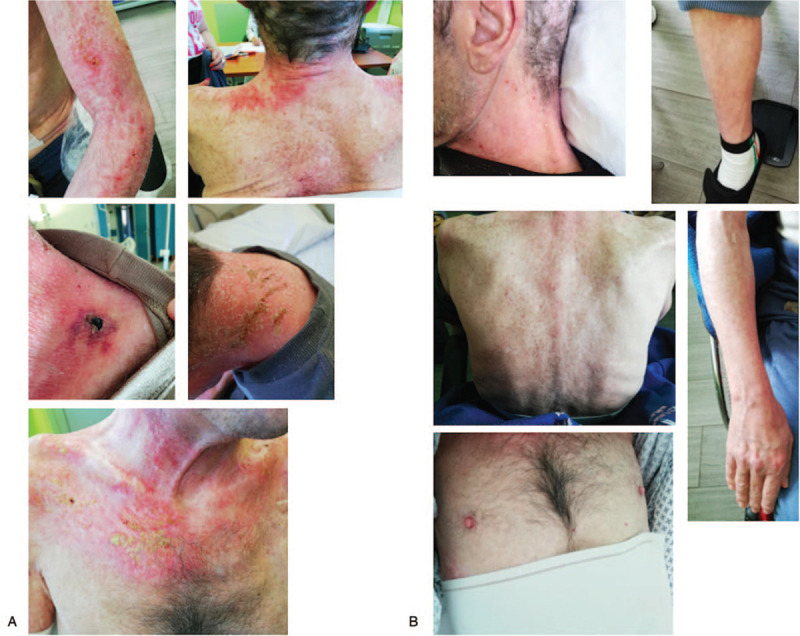
Cutaneous manifestations. Pictures of skin lesions before the first cycle of Begelomab (A) and skin soon after the end of the 5-day cycle of Begelomab (B).

After extensive discussion of the pros and cons with the patient, Ethical Committee approval and patient's written informed consent, MMF was discontinued and the anti-DPP-4 monoclonal antibody Begelomab was intravenously administered at the dosage of 4 mg/m^2^ daily for 5 consecutive days. At the end of the 5-day cycle of Begelomab, erythematous rash and skin ulcers almost completely healed (Fig. 4), the bloody diarrhea ceased, and C-reactive protein levels normalized. After 2 months, the patient experienced a further flare with the reappearance of high-grade fever, bloody diarrhea, and skin ulcers. A new 5-day cycle of Begelomab at the dosage of 4 mg/m^2^ daily was eventually administered (3 months and a half from the previous one). One week later, ileal perforation was recognized and the patient underwent emergency laparotomy with ileal resection. The postoperative course was complicated by anastomosis dehiscence and Candida peritonitis. A revision surgery was performed and, after resolution of the septic event, due to the persistence of significant diarrhea, the patient was treated with IVIG and a new 5-day cycle of Begelomab 4 mg/m^2^ daily followed by maintenance therapy with Begelomab at the dosage of 4 mg/m^2^ every other day for additional eleven infusions (Fig. 1). The treatment accomplished a considerable clinical response few days after Begelomab initiation. Gastrointestinal symptoms resolved completely and skin ulcers healed. Although severe muscle weakness persisted, improvement in dysphagia allowed the patient to gradually start semi-solid oral feeding. In the follow-up period, no DM exacerbation occurred and the patient's quality of life improved dramatically (Fig. 1).

Unfortunately, 2 months later, liver transaminase levels were found to be elevated at routine blood tests and an acute hepatitis C viral infection was diagnosed. Anti-viral therapy was soon initiated but the patient's general conditions progressively worsened until he died of respiratory failure following an episode of aspiration pneumonia.

## 3 Discussion

DM is a chronic relapsing-remitting disorder with great variability in disease phenotype. Clinical presentations range from amyopathic forms with exclusive cutaneous involvement to severe multi-organ dysfunction causing life-threatening complications.^[30]^ Gastrointestinal vasculitis is a fearsome manifestation and should be always suspected in case of DM patients complaining of persistent diarrhea or abdominal pain.^[31]^ Prompt recognition is crucial and treatment with immunosuppressive agents should be initiated as soon as infectious etiologies have been excluded, in order to avoid intestinal perforation. Cutaneous manifestations may also be disabling when ulcers and intense itching predominate. Despite physiotherapy, the immune-mediated damage of skeletal muscle often results in muscle atrophy and sarcopenia, with the co-existence of dysphagia when pharyngeal muscles are affected. Besides dramatically impacting on quality of life, DM clinical manifestations may represent a serious risk to life. An effective control of disease activity is critical.

Refractoriness to therapy is relatively common among patients with DM. Because of the lack of consensus guidelines for treatment in refractory cases, strategies for non-responders are based on largely empirical evidence, mostly from small case series and ongoing clinical trials.^[32]^ Adequate management of severe cases represents a major medical challenge and may require surgical procedures which expose immunocompromised patients to a considerable infectious risk.^[31]^ Also, the perioperative vacancy of immunosuppressive therapies may result in disease exacerbations.

The skin and muscle biopsies of the presented patient were studied for DPP-4/CD26 expression. Results guided the therapeutic choice to administer Begelomab to the patient through compassionate use in agreement with the patient and his family. Begelomab is a murine IgG2b monoclonal antibody against DPP-4/CD26, which upon binding to the molecule on activated T cells induces internalization of the DPP-4/CD26-Begelomab complex and inhibition of DPP-4/CD26-mediated immune mechanisms, ultimately leading to impaired expansion of auto-reactive T cells, key players in tissue damage occurring in DM.^[13–16,21]^ Published data on the clinical use of Begelomab are limited to patients with acute GvHD and demonstrated encouraging efficacy with an acceptable safety profile.^[29]^ In our patient, the clinical response was striking and above expectations. Begelomab induced clinical remission in our DM patient with the disappearance of cutaneous lesions, resolution of intestinal vasculitis, and improvement of dysphagia. Disease flares in the presented patient were sudden and rapidly progressive. Due to the lack of previous experience of Begelomab use in IIM, the interval between drug administrations was arbitrarily established based on the clinical picture. The ileal perforation observed soon after the second cycle of Begelomab presumably reflects a delay in drug administration when high disease activity had already caused irreversible intestinal damage. The subsequent cycle, performed after ileal resection, effectively reversed the DM flare with the resolution of both skin and gastrointestinal manifestations, suggesting that the immune response is efficiently switched off by Begelomab, whatever its degree of activation.

## 4 Conclusion

The patient had a very severe and refractory form of DM with relapsing intestinal vasculitis which underwent complete remission upon Begelomab therapy. This is the first described case of a patient with IIM treated with the anti-DPP-4 monoclonal antibody. This therapy was effective, suggesting that Begelomab might represent a novel promising agent for the treatment of refractory DM.

## Acknowledgments

The authors wish to thank ADIENNE Pharma & Biotech for the kind provision of Begelomab.

## Author contributions

**Conceptualization:** Rebecca De Lorenzo, Stefano Previtali, Patrizia Rovere-Querini.

**Data curation:** Clara Sciorati, Antonella Monno, Silvia Cavalli, Francesco Bonomi, Stefano Tronci, Stefano Previtali.

**Investigation:** Rebecca De Lorenzo, Clara Sciorati, Antonella Monno, Silvia Cavalli, Francesco Bonomi, Patrizia Rovere-Querini.

**Methodology:** Stefano Tronci.

**Resources:** Rebecca De Lorenzo.

**Supervision:** Patrizia Rovere-Querini.

**Writing – original draft:** Rebecca De Lorenzo.

**Writing – review & editing:** Clara Sciorati, Antonella Monno, Silvia Cavalli, Francesco Bonomi, Stefano Tronci, Stefano Previtali, Patrizia Rovere-Querini.

## References

[1] Dalakas MC. Inflammatory muscle diseases. N Engl J Med 2015;373:393–4. 10.1056/NEJMc1506827 26200989

[2] Rosa Neto NS Goldenstein-Schainberg C. Juvenile dermatomyositis: review and update of the pathogenesis and treatment. Rev Bras Reumatol 2010;50:299–312. 21125166

[3] Wang IJ Hsu WM Shun CT. Juvenile dermatomyositis complicated with vasculitis and duodenal perforation. J Formos Med Assoc 2001;100:844–6. 11802528

[4] Mamyrova G Kleiner DE James-Newton L. Late-onset gastrointestinal pain in juvenile dermatomyositis as a manifestation of ischemic ulceration from chronic endarteropathy. Arthritis Rheum 2007;57:881–4. 17530691 10.1002/art.22782 PMC2099313

[5] Niizawa M Maie O Asanuma Y. Adult dermatomyositis with angiopathy and cecum perforation. Nihon Hifuka Gakkai Zasshi 1991;101:447–51. 1886278

[6] Suwa A Hirakata M Hama N. An adult case of dermatomyositis complicated with cecum perforation and panniculitis. Nihon Rinsho Meneki Gakkai Kaishi 1997;20:60–6. 9105166 10.2177/jsci.20.60

[7] Malmstrom V Venalis P Albrecht I. T cells in myositis. Arthritis Res Ther 2012;14:230. 23270751 10.1186/ar4116 PMC3674618

[8] Fasth AE Dastmalchi M Rahbar A. T cell infiltrates in the muscles of patients with dermatomyositis and polymyositis are dominated by CD28null T cells. J Immunol 2009;183:4792–9. 19752224 10.4049/jimmunol.0803688

[9] Lahoria R Selcen D Engel AG. Microvascular alterations and the role of complement in dermatomyositis. Brain 2016;139 (Pt 7):1891–903. 27190020 10.1093/brain/aww122

[10] Greenberg SA Pinkus JL Kong SW. Highly differentiated cytotoxic T cells in inclusion body myositis. Brain 2019;142:2590–604. 31326977 10.1093/brain/awz207

[11] Allenbach Y Solly S Gregoire S. Role of regulatory T cells in a new mouse model of experimental autoimmune myositis. Am J Pathol 2009;174:989–98. 19218348 10.2353/ajpath.2009.080422 PMC2665758

[12] Schiaffino S Pereira MG Ciciliot S. Regulatory T cells and skeletal muscle regeneration. FEBS J 2017;284:517–24. 27479876 10.1111/febs.13827

[13] Lambeir AM Durinx C Scharpe S. Dipeptidyl-peptidase IV from bench to bedside: an update on structural properties, functions, and clinical aspects of the enzyme DPP IV. Crit Rev Clin Lab Sci 2003;40:209–94. 12892317 10.1080/713609354

[14] Klemann C Wagner L Stephan M. Cut to the chase: a review of CD26/dipeptidyl peptidase-4's (DPP4) entanglement in the immune system. Clin Exp Immunol 2016;185:1–21. 26919392 10.1111/cei.12781 PMC4908298

[15] Ishii T Ohnuma K Murakami A. CD26-mediated signaling for T cell activation occurs in lipid rafts through its association with CD45RO. Proc Natl Acad Sci U S A 2001;98:12138–43. 11593028 10.1073/pnas.211439098 PMC59781

[16] Morimoto C Schlossman SF. The structure and function of CD26 in the T-cell immune response. Immunol Rev 1998;161:55–70. 9553764 10.1111/j.1600-065x.1998.tb01571.x

[17] Ohnuma K Yamochi T Uchiyama M. CD26 up-regulates expression of CD86 on antigen-presenting cells by means of caveolin-1. Proc Natl Acad Sci U S A 2004;101:14186–91. 15353589 10.1073/pnas.0405266101 PMC521134

[18] Soare A Gyorfi HA Matei AE. Dipeptidylpeptidase 4 as a marker of activated fibroblasts and a potential target for the treatment of fibrosis in systemic sclerosis. Arthritis Rheumatol 2020;72:137–49. 31350829 10.1002/art.41058

[19] Varin EM Mulvihill EE Beaudry JL. Circulating levels of soluble dipeptidyl peptidase-4 are dissociated from inflammation and induced by enzymatic DPP4 inhibition. Cell Metab 2019;29:320–34. e325. 30393019 10.1016/j.cmet.2018.10.001

[20] Worthen CA Cui Y Orringer JS. CD26 identifies a subpopulation of fibroblasts that produce the majority of collagen during wound healing in human skin. J Invest Dermatol 2020;140:2515–24. e3. 32407715 10.1016/j.jid.2020.04.010 PMC7655599

[21] Ohnuma K Hatano R Komiya E. A novel role for CD26/dipeptidyl peptidase IV as a therapeutic target. Front Biosci (Landmark Ed) 2018;23:1754–79. 29772527 10.2741/4671

[22] Zhao Y. CD26 in autoimmune diseases: the other side of “moonlight protein”. Int Immunopharmacol 2019;75:105757. 31357088 10.1016/j.intimp.2019.105757

[23] Preusse C Goebel HH Held J. Immune-mediated necrotizing myopathy is characterized by a specific Th1-M1 polarized immune profile. Am J Pathol 2012;181:2161–71. 23058368 10.1016/j.ajpath.2012.08.033

[24] Bettelli E Oukka M Kuchroo VK. T(H)-17 cells in the circle of immunity and autoimmunity. Nat Immunol 2007;8:345–50. 17375096 10.1038/ni0407-345

[25] Allenbach Y Chaara W Rosenzwajg M. Th1 response and systemic treg deficiency in inclusion body myositis. PLoS One 2014;9:e88788. 24594700 10.1371/journal.pone.0088788 PMC3942319

[26] Tournadre A Miossec P. Interleukin-17 in inflammatory myopathies. Curr Rheumatol Rep 2012;14:252–6. 22350607 10.1007/s11926-012-0242-x

[27] Morimoto C Torimoto Y Levinson G. 1F7, a novel cell surface molecule, involved in helper function of CD4 cells. J Immunol 1989;143:3430–9. 2479677

[28] Salgado FJ Perez-Diaz A Villanueva NM. CD26: a negative selection marker for human Treg cells. Cytometry A 2012;81:843–55. 22949266 10.1002/cyto.a.22117

[29] Bacigalupo A Angelucci E Raiola AM. Treatment of steroid resistant acute graft versus host disease with an anti-CD26 monoclonal antibody-Begelomab. Bone Marrow Transplant 2020;55:1580–7. 32203257 10.1038/s41409-020-0855-z

[30] DeWane ME Waldman R Lu J. Dermatomyositis: clinical features and pathogenesis. J Am Acad Dermatol 2020;82:267–81. 31279808 10.1016/j.jaad.2019.06.1309

[31] Villa R Costa S Focchi S. Successful open abdomen treatment for multiple ischemic duodenal perforated ulcers in dermatomyositis. World J Emerg Surg 2014;9:48. 26085838 10.1186/1749-7922-9-48 PMC4470353

[32] Patwardhan A Spencer CH. Biologics in refractory myositis: experience in juvenile vs. adult myositis; part II: emerging biologic and other therapies on the horizon. Pediatr Rheumatol Online J 2019;17:56. 31429786 10.1186/s12969-019-0361-2 PMC6702719

